# Perinatal BPA Exposure Induces Hyperglycemia, Oxidative Stress and Decreased Adiponectin Production in Later Life of Male Rat Offspring

**DOI:** 10.3390/ijerph110403728

**Published:** 2014-04-03

**Authors:** Shunzhe Song, Ling Zhang, Hongyuan Zhang, Wei Wei, Lihong Jia

**Affiliations:** Department of Child and Adolescent Health, School of Public Health, China Medical University, 92 North 2nd Road, Shenyang 110001, China; E-Mails: songsz_cmu@163.com (S.S.); zhangling_19880808@163.com (L.Z.); 13889305978@163.com (H.Z.); weiwei840403@163.com (W.W.)

**Keywords:** bisphenol A, glucose metabolism, adiponectin, oxidative stress, puberty stage, adult stage

## Abstract

The main object of the present study was to explore the effect of perinatal bisphenol A (BPA) exposure on glucose metabolism in early and later life of male rat offspring, and to establish the potential mechanism of BPA-induced dysglycemia. Pregnant rats were treated with either vehicle or BPA by drinking water at concentrations of 1 and 10 µg/mL BPA from gestation day 6 through the end of lactation. We measured the levels of fasting serum glucose, insulin, adiponectin and parameters of oxidative stress on postnatal day (PND) 50 and PND100 in male offspring, and adiponectin mRNA and protein expression in adipose tissue were also examined. Our results showed that perinatal exposure to 1 or 10 µg/mL BPA induced hyperglycemia with insulin resistance on PND100, but only 10 µg/mL BPA exposure had similar effects as early as PND50. In addition, increased oxidative stress and decreased adiponectin production were also observed in BPA exposed male offspring. Our findings indicated that perinatal exposure to BPA resulted in abnormal glucose metabolism in later life of male offspring, with an earlier and more exacerbated effect at higher doses. Down-regulated expression of adiponectin gene and increased oxidative stress induced by BPA may be associated with insulin resistance.

## 1. Introduction

Bisphenol A (BPA), a base compound in the production of polycarbonate plastic, is a kind of potential endocrine disruptor. The human population is continuously and inevitably exposed to low doses of BPA in daily life [1,2]. Importantly, BPA has been detected in amniotic fluid, cord blood and human breast milk, which demonstrates the potential of this compound to pass from mother to fetus [3]. Evidence suggests that conditions experienced during early development play an important role in determining the long-term health of individuals [4]. Animal experiments showed that prenatal BPA exposure resulted in abnormal glucose metabolism in future life [5,6]. Human study also found that the levels of urinary BPA increase in patients with type 2 diabetes [7,8]. The mechanism of BPA interference with glucose metabolism is not fully clear, but estrogenic effects are implicated. Increasing evidence shows that adverse effects of BPA on health are miscellaneous, varying with duration, doses and route of BPA exposure, as well as sex difference. 

BPA is able to mimic estrogen activity, and increase insulin secretion from β-cells in pancreas [9,10]. In addition, it can also interfere with insulin transduction of target cells, inducing insulin resistance [11]. Recently, two important factors related to BPA have been highlighted for its relationship with insulin resistance, namely oxidative stress and adiponectin production [12,13]. 

BPA induces dysfunction of insulin secretion through the damage of mitochondria in rat insulinoma (INS-1) cells [12]. BPA results in hepatotoxicity of rat by generating reactive oxygen species (ROS) [14]. Mitochondrial dysfunction induced by oxidative stress plays an important role in the occurrence of insulin resistance [15]. Study on the hyperglycemia and oxidative stress induced by BPA at same time has not been reported until now, especially in male rats. Malondialdehyde (MDA) is an end product of lipid peroxidation, which is used as an indicator of oxidative stress in cells and tissues [16]. The higher are MDA levels, the more serious oxidative injuries represent. Superoxide dismutase (SOD) is a class of enzymes which catalyses the dismutation of superoxide into oxygen and hydrogen peroxide. SOD activity is usually lower with the increased oxidative stress, so serum MDA and SOD levels are two typical parameters of oxidative stress. 

In addition, the reduction of adiponectin (ADP) is another important factor related to insulin resistance. It has been reported that BPA inhibits ADP production and secretion in 3T3-L1 adipocytes through down-regulation of Akt signaling [13]. As a crucial adipokine, ADP can enhance insulin sensitivity by binding to its receptors, eliciting the activation of insulin signal pathway such as AMPK [17]. In addition, ADP also promotes the combustion of fatty acid and triglyceride in skeletal muscle [18]. With the inhibition of ADP release induced by BPA, the content of free fatty acid (FFA) and triglyceride increases, leading to insulin resistance [19]. Based on accumulating evidence, researchers have revealed that perinatal BPA exposure has a profound adverse effect on glucose metabolism in future life. However, very few experiments have been conducted to investigate differential effects BPA exerted at different life stages as a result of perinatal exposure, especially in male rats. In the present study, male rats were exposed to different doses of BPA during early development, and their body weight, the levels of serum glucose, insulin, ADP and parameters of oxidative stress on PND50 or PND100 were measured respectively. In addition, the levels of ADP mRNA and protein expression from subcutaneous adipose tissue were also determined on PND100. Our findings demonstrated that low or high doses of BPA (1 or 10 µg/mL) may induce insulin resistance on adult stage, but high doses of BPA induce earlier occurrence of hyperglycemia and exacerbate symptom. Insulin resistance induced by BPA is associated with decreased ADP production and increased oxidative damage. 

## 2. Materials and Methods

### 2.1. Animal Treatment

Sprague-Dawley (SD) female (200–220 g body weight) and male (250–300 g body weight) rats were purchased from the animal center of China Medical University. All the animals were handled in accordance with the Guidelines for Animal Experimentation issued by the Chinese Association for Laboratory Animal Science. Both water bottles and polypropylene cages were devoid of BPA. After a 1-week adaptation period in a room with standard temperature (22 ± 2 °C) and illumination (12 h light-dark), females were mated with males. A sperm-positive vaginal smear was taken to indicate the first day of pregnancy. Pregnant rats of normal physiological condition were housed individually and randomly allocated into three groups (*n* = 7/group). Two groups were exposed to BPA (Sigma-Aldrich Chemical Co. St. Louis, MO, USA) by free access to water at levels of 1 µg/mL (F0-BPA1) and 10 µg/mL (F0-BPA10) respectively from gestation day 6 through the end of lactation. The third group, as control group (F0-control) was given water containing 1% ethanol, the concentration used as vehicle for BPA solution. Maternal food intake and water consumption were measured daily during gestation and lactation. We declared the first day of parturition as postnatal day 0 (PND0), and offspring from F0-control, F0-BPA1 and F0-BPA10 as F1-control, F1-BPA1 and F1-BPA10 respectively. Maternal rats with their offspring were pooled together and placed in different litters belonging to each group respectively. From PND 21, the end of lactation, male offspring were given normal water without BPA and fed with standard chow diet until their adult stage (PND100). The female offspring were used for other study. Water consumption was measured daily according to the volume reduction of the bottles, and body weight was recorded weekly for male offspring. At puberty stage (PND50) and adult stage, seven male offspring belonging to seven different litters were randomly chosen from each group respectively. Then we performed the following assay to investigate the effects of BPA on glucose metabolism, oxidative stress and ADP production in blood or subcutaneous fat tissue at puberty and adult stage respectively.

### 2.2. Histologic Examination of Subcutaneous Tissue Oil Red O

At their adult stage, three male offspring were randomly selected from each independent group respectively. We killed them immediately and dissected their subcutaneous adipose tissues. Adipose tissues were fixed in paraformaldehyde solution, and then embedded in paraffin. Sections were cut with the facility of microtome (Leica Microsystems, Werzlar, Germany) and stained with oil red O. Photos were taken using the microscope with photographical arrangement (Leica Microsystems). 

### 2.3. Blood and Tissue Collection

At their puberty stage, 21 male offspring in total were randomly selected from the three groups (*n* = 7/group). After an overnight fasting period, offspring were anesthetized with ether for alleviation of suffering. Immediately we obtained their blood samples from abdominal aorta and dissected their subcutaneous adipose tissue. Serum was separated by centrifugation and stored at −80 °C for subsequent analysis of serum glucose, insulin, ADP and parameters of oxidative stress. Subcutaneous adipose tissues were collected and stored at −80 °C for later use in measuring the expression of ADP protein and mRNA. Similarly, when other offspring grew up to adult stage, the same procedure was conducted as described above to assay the alteration of following parameters. 

### 2.4. Biochemical Assays

Glucose metabolism was evaluated by the level of serum glucose and insulin. Serum glucose level was measured by using a glucometer (OneTouch Ultra). Serum insulin concentration was measured by insulin radioimmunoassay kits (Beijing Atom High Tech Co., Ltd. Beijing, China) according to manufacturer’s protocol. A Homeostatic Model Assessment for Insulin Resistance (HOMA-IR index) was calculated by blood glucose (mmol/L) × insulin (mU/L)/22.5 [20].

Serum ADP level was measured by ELISA kits (R&D System China Co. Ltd., Shanghai, China) according to the manufacturer’s instructions. We measured the level of MDA by using a commercial MDA kit (Nanjing Jianchen Bioengineering Company, China). The principle of the method is based on the spectrophotometric measurement of the color produced during the reaction of thiobarbituric acid (TBA) with MDA. Concentration of TBA-reactive substances was calculated by the absorbance coefficient of malondialdehyde-TBA complex and expressed as unit of nmol/mL serum. Similarly the activities of SOD and Total anti-oxidant capacity (T-AOC) were measured with assay kit (Nanjing Jianchen Bioengineering Company, Nanjing, China) according to the manufacturer’s instructions. SOD and T-AOC were expressed as unit of activity per mL serum (U/mL). 

### 2.5. Western Blotting

The level of ADP protein expression in subcutaneous adipose tissues was measured by western blotting. Protein samples were prepared from subcutaneous adipose tissues, and its concentration was determined by Pierce BCA Protein Assay Kit (Thermo Scientific, Rockford, IL, USA). Samples containing 50 μg of protein were mixed with 5 × SDS -acrylamide gels loading buffer of equal volumes, and incubated at 95 °C for 5 min. Each sample of 20 μL aliquots was loaded onto 14% SDS-acrylamide gels. Proteins were separated by application of a constant voltage of 100 V for 1.5 h. 

For immunodetection of ADP, the membranes were blocked overnight at 4 °C with TBS containing 0.1% Tween 20 and 5% nonfat milk, and incubated overnight at 4 °C with anti-ADP primary antibodies: monoclonal mouse anti human ADP (1:1000; sc-26496; Santa Cruz Biotechnology, Santa Cruz, CA, USA) in TBSTM. The membranes were then incubated for 1 h using rabbit anti mouse IgG (1:5000; Santa Cruz) secondary antibodies conjugated with horseradish peroxidase in TBSTM. A similar process was also performed to assay GAPDH as an internal reference. Autoradiograph was scanned by densitometry to quantify differences and analyzed by using Phoretix 1D advanced software. The signal intensity of each ADP band was normalized to the corresponding GAPDH band. Five samples from each group were performed. 

### 2.6. Real-Time PCR

Total RNA was extracted from subcutaneous adipose tissue using TRIzol (Takara Biotechnology, Dalian, China). Total RNA with A260:A280 ratio above 1.85 was used for real-time PCR analyses. First strand cDNA was reverse-transcribed from 1 μg of total RNA by using a SuperScript first-strand synthesis kit (Takara) according to the manufacturer’s instruction. Quantification of murine ADP and β-actin mRNA was performed with a PRISM 7700 sequence detector (Life Technologies, Shanghai, China), by using TaqMan probe. The primers were designed by Takara Biotechnology. PCR cycling conditions were as follows: 2 min at 50 °C, 10 min at 95 °C followed by 40 cycles of denaturation at 95 °C for 15 s, and annealing and extension at 60 °C for 1 min. Amplification was performed in a final volume of 25 µL, and each sample contained 3 µL cDNA (equivalent to 100 ng of RNA), 900 nmol of specific primers, 225 nmol of the probe, and a master mix made from quantitative PVR core kit (Takara). The mean value of Ct for each sample was used for data analysis. All samples were normalized to the β-actin values and the results expressed as fold changes of Ct changes of Ct value relative to controls by using the 2^−ΔΔ Ct^ formula. 

### 2.7. Statistical Analysis

All analysis was performed with SPSS 11.5 software (SPSS, Inc., Chicago, IL, USA). The data was expressed as mean ± SE. Differences between groups were analyzed by LSD of post-hoc after one-way ANOVA. *p* < 0.05 was considered statistically significant. 

## 3. Results

### 3.1. Maternal Physiology during Gestation

To evaluate whether BPA exposure during gestation affected maternal physiology, we measured the change of body weight and the amount of food and water intake during pregnancy in dams BPA- exposed. The change of body weight throughout the pregnancy was no significant difference in the BPA-exposed dams and control dams (*p* > 0.05). Average amount of food intake and volume of water consumed daily at low or high BPA-exposed dams were similar as control dams during pregnancy (Table 1). 

### 3.2. Effect of BPA Exposure on Body Weight in Male Offspring from PND1 to PND100

To examine whether perinatal BPA exposure predisposes an adverse effect on glucose metabolism in future development. We studied three groups of male rats: F1-Control, F1-BPA1 and F1-BPA10. Note that these offspring received no direct BPA treatment, only their mothers received BPA treatment with different doses.

**Figure 1:**
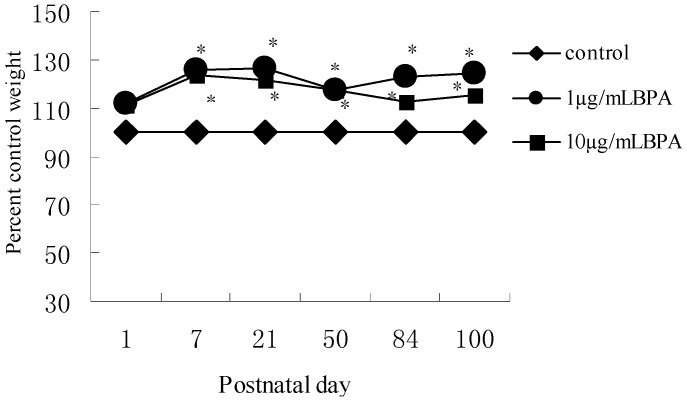
Effect of perinatal exposure BPA on body weight from postnatal day 1 (PND1) to PND100 in male offspring. Pregnant rats (*n* = 7/group) were exposed to vehicle or BPA in water at levels of 1 or 10 µg/mL from gestation day 6 through the end of lactation. Values represent the mean ± SE (*n* = 7, one from each group). * *p* < 0.05 compared with control.

We recorded mean body weight of three independent groups from birth to adult stage (*n* = 7, each pup from per litter of each group). The body weight of BPA treated groups was significantly heavier than that of control group on PND7. Increased body weight remained until adult stage (PND100) without statistical significance between F1-BPA1 and F1-BPA10 (Figure 1). These results suggested the effect of low doses of perinatal BPA exposure on body weight was more significant than that of high doses of BPA in later life. 

**Table 1:** Effects of BPA exposure during pregnancy and lactation on maternal physiology. Pregnant rats (*n* = 7/group) were exposed to vehicle or BPA in water at levels of 1 or 10 µg/mL from gestation day 6 through the end of lactation. The change of body weight and the amount of food and water intake during pregnancy were measured. Values represent the mean ± SE (*n* = 7, one from each group) by one-way ANOVA.

	F0-Control	F0-BPA1	F0-BPA10
Body weight on GD 0 (g)	262.7 ± 9.58	258.8 ± 6.49	251.3 ± 6.81
Body weight gain (g)			
GD 0–7	25.3 ± 3.1	23.6 ± 1.3	25.2 ± 1.9
GD 8–14	27.6 ± 4.7	29.9 ± 4.6	28.4 ± 2.9
GD 15–21	55.9 ± 8.2	60.0 ± 4.8	59.6 ± 6.4
Food consumption (g per day)			
GD 0–7	24.9 ± 3.4	27.6 ± 1.5	24.7 ± 1.2
GD 8–14	22.5 ± 4.3	28.3 ± 3.4	25.9 ± 4.9
GD 15–21	25.4 ± 4.1	26.0 ± 3.7	23.6 ± 1.6
Water consumption (mL per day)			
GD 0–7	33.8 ± 2.3	33.6 ± 2.6	27.7 ± 1.8
GD 8–14	33.2 ± 4.9	35.8 ± 4.8	29.4 ± 2.4
GD 15–21	20.2 ± 2.1	25.0 ± 2.3	25.6 ± 2.9

### 3.3. Effect of BPA Exposure on Glucose Metabolism of Male Offspring at Puberty Stage and Adult Stage

**Figure 2:**
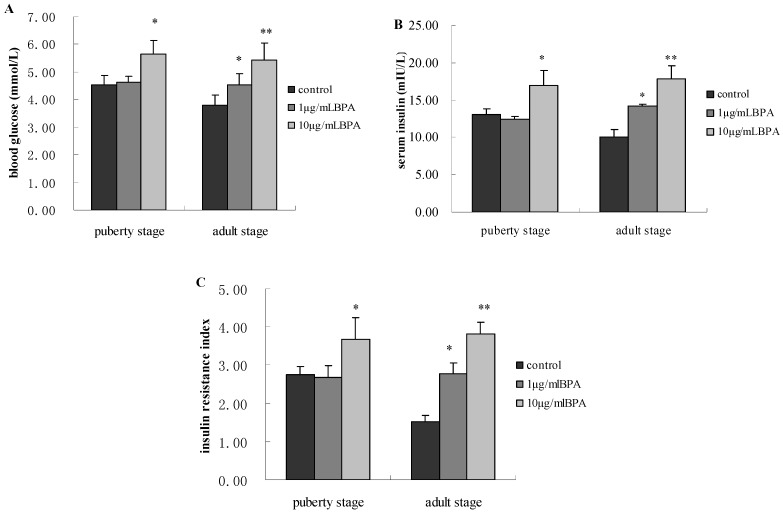
Effects of perinatal exposure to BPA on glucose metabolism of male offspring at their puberty stage and adult stage. Pregnant rats (*n* = 7/group) were exposed to vehicle or BPA in water at levels of 1 or 10 µg/mL from gestation day 6 through the end of lactation. Serum glucose level was measured by using a glucometer (OneTouch Ultra). Serum insulin concentration was measured by insulin radioimmunoassay kits. HOMA-IR = fasting insulin (μIU/mL) × glucose (mmol/L)/22.5. Values represent the mean ± SE (*n* = 7, one from each group). * *p* < 0.05 and ** *p* < 0.01 compared with control.

We assayed the levels of serum glucose and insulin in male offspring at pubertal stage. For F1-BPA1, there was no significant change in the two parameters compared with control (*p* > 0.05). As for F1-BPA10 male rats, BPA significantly increased their blood glucose and insulin (*p* < 0.05). When they reached adult stage, we observed that the two parameters were significantly increased in both F1-BPA1 and F1-BPA10 groups (*p* < 0.05 or *p* < 0.01). In addition, the two parameters were significantly higher in F1-BPA10 male rats compared with F1-BPA1 (*p* < 0.05, Figure 2A,B). 

We used the formula mentioned above to calculate the insulin resistance index. For F1-BPA1, insulin resistance did not increase significantly compared with control until adult stage. And for F1-BPA10, insulin resistance started to increase significantly from puberty stage (*p* < 0.05). At adult stage, insulin resistance of F1-BPA10 was higher than that of F1-BPA1 with statistical significance (*p* < 0.05, Figure 2C). 

### 3.4. Effect of BPA Exposure on ADP Production and Release in Male Offspring at Puberty Stage or Adult Stage

**Figure 3:**
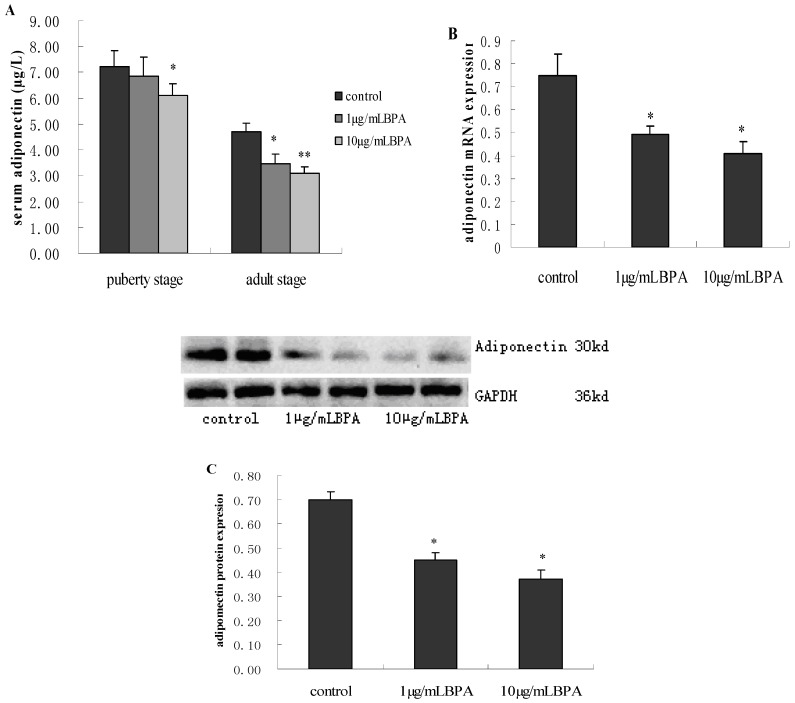
Effects of perinatal exposure to BPA on adiponectin (ADP) production from blood or subcutaneous adipose tissue of male offspring at their puberty and adult stage. Pregnant rats (*n* = 7/group) were exposed to vehicle or BPA in water at levels of 1 or 10 µg/mL from gestation day 6 through the end of lactation. Serum ADP level was measured by ELISA kits (**A**) The levels of adiponectin mRNA (**B**) and protein (**C**) expression in subcutaneous adipose tissue were measured by Real-time PCR and western blotting respectively. Values represent the mean ± SE (*n* = 7, one from each group). * *p* < 0.05 and ** *p* < 0.01 compared with control.

Following a similar pattern of insulin resistance, 1 µg/mL BPA treatment did not significantly decrease the level of serum ADP until adult stage. While 10 µg/mL BPA treated group showed decreased ADP levels as early as their puberty stage compared with control. However, the difference in serum ADP levels between 1 µg/mL BPA and 10 µg/mL BPA was not of statistical significance (*p* > 0.05, Figure 3A). Therefore, we performed subsequent assay on the expression of ADP mRNA and protein from subcutaneous adipose tissue only at adult stage. 

For BPA treated groups, there were statistically significant decrease in the levels of ADP mRNA and protein compared with control group (*p* < 0.05). Nevertheless, the slight decrease in F1-BPA10 compared with F1-BPA1 was not statistically significant, which is consistent with our result in serum ADP levels at adult stage (Figure 3B,C). 

### 3.5. Effect of BPA Exposure on the Size of Subcutaneous Adipocytes of Male Offspring at Adult Stage

**Figure 4:**
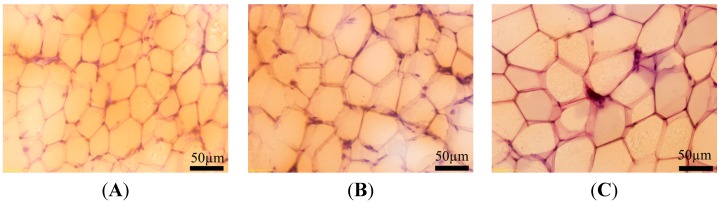
Effect of perinatal exposure to BPA on morphological difference of subcutaneous adipocytes for male offspring at adult stage. Pregnant rats (*n* = 7/group) were exposed to vehicle or BPA in water at levels of 1 or 10 µg/mL from gestation day 6 through the end of lactation. Histological sections of subcutaneous adipocytes with Oil red O were made in male offspring at adult stage from each group. Image (**A**–**C**): Representative histological sections of subcutaneous adipose tissue from F1-control, F1-BPA1 and F1-BPA 10 respectively.

In order to investigate the effect of perinatal exposure to 1 or 10 µg/mL BPA on morphological volume of adipocytes, which are related to body ADP releases. We made histological sections of subcutaneous adipocytes with the rats of adult stage from each group respectively. Clearly, the size of adipocytes treated with BPA is bigger than that of control. There was no apparently different in adipocytes size of F1-BPA1 compared with F1-BPA10 (Figure 4). 

### 3.6. Effect of BPA Exposure on Serum SOD, MDA and T-AOC in Male Offspring at Puberty Stage or Adult Stage

**Figure 5:**
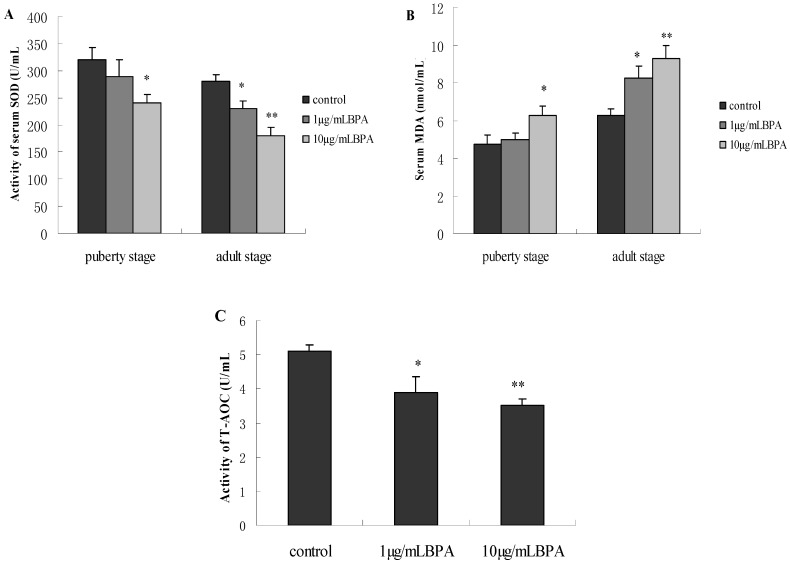
Effects of perinatal exposure to BPA on the parameters of oxidative stress. Pregnant rats (*n* = 7/group) were exposed to vehicle or BPA in water at levels of 1 or 10 µg/mL from gestation day 6 through the end of lactation. The levels of serum SOD, MDA and T-AOC were measured by a commercial kit. Values represent the mean ± SE (*n* = 7, one from each group). * *p* < 0.05 and ** *p* < 0.01 compared with control.

At puberty stage, the activity of serum SOD was significantly lower, and the levels of serum MDA was significantly higher in F1-BPA10 compared with controls (*p* < 0.05). There was no significant difference in SOD and MDA between F1-BPA1 and controls (*p* > 0.05). But at adult stage, there were significantly dose-dependent decrease in the activity of SOD and increase in MDA levels (*p* < 0.05). In addition, the difference in SOD and MDA between F1-BPA1 and F1-BPA10 was statistically significant (Figure 5). 

We further measured the effect of BPA on serum total antioxidant ability (T-AOC), and observed a dose-dependent decrease in the activity of serum T-AOC for male offspring at adult stage, which is in accordance with SOD (Figure 5). Our findings show that perinatal BPA exposure induces oxidative stress of male offspring in later life. 

## 4. Discussion

In our study, pregnant rats were exposed to 1 µg/mL or 10 µg/mL BPA in water from day 6 of gestation to the end of lactation. This was the most likely route of human exposure. The average daily volume of water consumed was no significant difference between control dams (31 ± 6 mL) and BPA-exposed dams (33 ± 7 mL for F0-BPA1 and 31 ± 9 mL for F0-BPA10) during pregnancy. Based on these measurements, we estimated that mean levels of BPA consumed daily by pregnant females was approximately 33 μg and 310 μg BPA/dam during gestation in F0-BPA1 and F0-BPA10 respectively. As gestating dam average weighed approximately 412 ± 14 g for F0-BPA1 and 409 ± 10 g for F0- BPA10 before parturition, we counted the total BPA exposure to be approximately 80 and 758 μg/kg/day in F0-BPA1 and F0-BPA10 respectively during pregnancy. Bottle leakage or spillage could result in actual BPA exposure being lower than our calculated levels. The doses of BPA we tested here were considered as a low or high BPA exposure according to related reference [21]. The amount of food intake in BPA-exposed dams was no different compared with controls (Table 1). These findings indicated that 1 µg/mL or 10 µg/mL BPA in water had no significant effect on maternal weight gain, food intake and water consumed during pregnancy. 

It is well known that conditions experienced in embryo can have lifelong effects on health, according to the so-called “fetal plasticity” theory [4]. Thus, we did the present study with male rat offspring to investigate the effects of perinatal BPA exposure on glucose metabolism at different developmental stages, and to evaluate the change in the levels of adiponectin and oxidative stress, two factors closely related with glucose metabolism. Our findings showed that perinatal BPA exposure led to hyperglycemia accompanied by insulin resistance in future development of male offspring, in consistent with the study by Alonso-Magdalena [5]. Focusing on glucose metabolism, 1 µg/mL BPA treatment resulted in adverse effect on insulin sensitivity until adult stage, while 10 µg/mL BPA treated group had similar effect earlier (Figure 2). Based on the observation above, we made the conclusion: with our experimental range of BPA, the more doses exposed perinatally, the earlier BPA made its adverse effect on glucose metabolism. No matter low or high doses of BPA exposed during early development, it has long-term adverse effect on glucose metabolism in later life. 

ADP, as a vital kind of adipokines, is mainly expressed and released from adipose tissue. It is important to note that increasing tissue triglyceride and free fatty acid (FFA) interferes with insulin-stimulated activation of phosphatidylinositol 3-kinase (PI3-K) and subsequent glucose transporter translocation, leading to insulin resistance [17]. ADP has been confirmed to increase the combustion of FFA and triglyceride and improve insulin signal transduction [17]. A recent study found that BPA at environmentally relevant doses inhibits ADP release from human adipose tissue explants and adipocytes [22]. In the present study, ADP production of BPA-treated male offspring was significantly lower than control, whether from blood or subcutaneous adipose tissue (Figure 3). In addition, histological sections demonstrated that the size of adipocytes was obviously large in BPA-exposed rats than that in controls at adult stage (Figure 4). Histological sections indirectly highlighted the decrease in ADP release, supported by the theory: adipocytes of bigger size suppress ADP release. Based on the decrease in ADP release in parallel with insulin sensitivity, we speculate that decreased ADP induced by BPA may be a critical factor in mediating hyperglycemia. 

In our study, the effect of BPA on oxidative stress was similar to ADP response. Study showed that an increase in glucose concentrations can lead to tissue damage by increasing oxidative stress [23]. Oxygen free radical decreases insulin sensitivity by interfering with tyrosine phosphorylation of insulin receptor substrate-1 (IRS-1) [24]. In the present study, we only observed a significantly increased serum MDA levels and decreased serum SOD activity in high doses of BPA-exposed offspring at puberty stage, not in low doses of BPA-treated offspring. The both of serum MDA and SOD levels were lower at adult stage, no matter in lower or higher dose of BPA-treated offspring. There was a dose-dependent decrease in insulin sensitivity in parallel with the activity of serum T-AOC at adult stage. These findings showed that the changes of insulin resistance, ADP production and oxidative stress induced by BPA exposure were concordant. The observation suggested that BPA-induced insulin resistance is also associated with oxidative stress. However, the evidence is not enough to prove that BPA-induced oxidative damage is due to the outcome hyperglycemia, rather than the direct effect of BPA. 

Recently, multiple studies in human and rodents had found that prenatal and early postnatal BPA exposure was associated with increased body weight [25,26]. The outcomes here are in line with these findings. It is well known that obesity increases the risk of insulin resistance and oxidative stress. In our present study, perinatal BPA exposure induced significantly high body weight in male offspring from born to adulthood. We found that there was no statistically significant difference between F1-BPA1 and F1-BPA10 before puberty. But on PND84 and PND100, body weight in F1-BPA1 was higher than that in F1-BPA10, and the result was similar to the change in morphology of subcutaneous adipocytes as described above. In our study, the change in ADP production induced by low or high doses of BPA was mainly in line with the level of insulin resistance. While, the severity of oxidative stress induced by low or high doses of BPA was in parallel with the levels of insulin resistance in adult stage. Our results suggested perinatal BPA exposure had a serious adverse effect on body weight and insulin resistance in later life, which was associated with decreased adiponectin production and increased oxidative stress. 

In recent years, the effect of BPA exposure on adipocytes attracts broad attention. Numerous *in vitro* studies have shown that BPA interferes with glucose homeostasis in 3T3-F442A adipocytes by increasing basal and insulin-stimulated glucose uptake [27], and promotes 3T3-L1 fibroblasts to differentiate into adipocytes [28]. BPA decreased adiponectin release from human adipose tissue [19]. Perinatal exposure to a low dose of BPA increased adipogenesis in females at weaning by increasing mRNA levels of fatty acid synthase (FAS) in rats [26]. Low doses of BPA interfere with inflammatory/insulin signaling pathways, leading to impairment of adipose cell function [29]. These studies combined with our findings imply that BPA interferes with the metabolism and function of adipocyte, which increased the risk of related diseases such as obesity, type 2 diabetes and cardiovascular and cerebrovascular diseases. 

## 5. Conclusions

The “developmental” origin of adult disease hypothesis states that environmental factors act in early life and programs the risks of developing chronic disease in future life [30]. Our findings in the present study indicated that perinatal exposure to low or high doses of BPA induces abnormal glucose metabolism and insulin resistance in later life. Exposure at higher doses may cause earlier occurrence of hyperglycemia and aggravate the symptom, and insulin resistance induced by BPA was associated with decreased ADP production and increased oxidative damage. Based on our experiments with SD rats, we advise pregnant women to keep away from plastic products containing BPA during gestation and lactation to decrease future risk of obesity and hyperglycemia for themselves and their children. 
